# Role of mucin 2 gene for growth in *Anas platyrhynchos*: a novel report

**DOI:** 10.3389/fvets.2023.1089451

**Published:** 2023-11-09

**Authors:** Anuj Kumar Murmu, Aruna Pal, Manti Debnath, Argha Chakraborty, Subhamoy Pal, Samiddha Banerjee, Abantika Pal, Nilotpal Ghosh, Utpal Karmakar, Rajarshi Samanta

**Affiliations:** ^1^Department of Livestock Production and Management, West Bengal University of Animal and Fishery Sciences, Kolkata, West Bengal, India; ^2^Department of Livestock Farm Complex, West Bengal University of Animal and Fishery Sciences, Kolkata, West Bengal, India; ^3^Department of Animal Science, Visva-Bharati University, Santiniketan, West Bengal, India; ^4^Department of Computer Science, Indian Institute of Technology Kharagpur, Kharagpur, West Bengal, India; ^5^University of California, San Francisco, San Francisco, CA, United States; ^6^Department of Animal Resource Development, Government of West Bengal, Kolkata, India

**Keywords:** indigenous duck, mucin, body weight, biomorphometric characteristics, differential mRNA expression profiling

## Abstract

**Introduction:**

The mucin gene is expressed in the mucous membrane of the inner layer of the internal organs. Intestinalmucin 2 (MUC2), amajor gel-formingmucin, represents a primary barrier component of mucus layers.

**Materials and methods:**

This is the first report on the role of mucin genes in growth traits in animals. In this study, we randomly studied Bengal ducks (Anas platyrhynchos) reared from day old to 10 weeks of age under an organized farm and studied the growth parameters as well as body weight and average daily body weight gain.

**Result and discussion:**

We characterized the mucin gene for Bengal ducks and observed glycosylation and EGF1 (EGF-like domain signature) as important domains for growth traits in ducks. We observed a better expression profile for the mucin gene in high-growing ducks in comparison to that of low-growing ducks with real-time PCR. Hence, the mucin gene may be employed as a marker for growth traits.

## Introduction

Mucus is a viscous gel-like material covering the gastrointestinal mucosal surface. The entire surface of the chicken gastrointestinal tract is covered by a layer of mucus that functions as a diffusive barrier between the intestinal lumen and absorptive cells. The mucins are the main component of the mucus layer, which are produced and secreted by goblet cells. Mucins are high-molecular weight glycoproteins (50–80% O-linked oligosaccharides) produced by epithelial tissues in most animals. These glycoproteins are found in mucus (e.g., saliva and gastric juice) and secreted by mucous membranes to lubricate or protect body surfaces of vertebrates, and they have a central role in maintaining epithelial homeostasis (1). The mucus layer is part of the innate host response, protecting against luminal microflora, preventing gastrointestinal pathologies, and participating in the processes of nutrient digestion and absorption (2). A decrease in mucin synthesis in poultry could compromise the mucus layer and reduce nutrient utilization (3).

Mucins are divided into secretory and membrane-bound types based on their forms. Membrane-bound mucins (MUC1, MUC3A, MUC3B, MUC4, MUC12, MUC13, MUC15, MUC16, MUC17, MUC20, and MUC21) exhibit hydrophobic sequences or “transmembrane domains” responsible for anchoring them in the lipid bilayer and have C-terminal peptides that enter the cytosol. The secretory mucins (MUC2, MUC5AC, MUC5B, MUC6, MUC8, and MUC19) with one exception (MUC7) possess one or several von Willebrand factor (vWF)-like D domains and cysteine-rich peptides, which function in the oligomerization of mucin monomers and in packaging into secretory vesicles (4–9). Based on whether they are capable of forming a gel, the secretory mucins can be further divided into two subtypes, namely, gel-forming and soluble MUCs. Interestingly, MUC2, MUC5AC, MUC5B, and MUC6 belong to gel-forming MUCs and are located close to each other on human chromosome 11p15.5 (10).

Mucin is the major constituent of the mucus layer and serves a crucial role in protecting the gut from acidic chyme, digestive enzymes, and pathogens. In addition to its protective functions, mucin is involved in filtering nutrients in the gastrointestinal tract (GIT) and can influence nutrient digestion and absorption (11). Any component, dietary or environmental, that induces changes in mucin dynamics has the potential to affect viscosity, integrity of the mucus layer, and nutrient absorption. Mucins in general contain many threonine and serine residues, which are extensively O-glycosylated. Due to this profound glycosylation, mucins have a filamentous conformation.

Ducks were observed to be mostly foragers with better disease resistance (12), higher adaptability, and hardy breed (13, 14). Apart from metagenomic studies, the gut of the foraging birds forms a major role in providing immunity and the hardy nature of the birds, particularly ducks raised under semi-intensive system of management. Although reports were available for the role of mucin in nutrient absorption and digestion, no report as such was available for the role of the mucin gene in growth traits at a molecular level. Growth trait is important for both the meat and egg production of the duck. We had earlier studied the importance of certain genes in the growth of livestock (15–19). Growth is a polygenic quantitative trait, and hence it is important to study certain genes, like mucin gene, affecting gut integrity and nutrient absorption scenario. In our lab, we are studying growth parameters including body weight and growth parameters for livestock species (13, 15–21). Hence, this study was designed to assess the role of mucin gene in the growth of ducks through differential mRNA expression profiling.

## Materials and methods

### Birds

This study was conducted on Bengal ducks under organized farming system. This investigation was carried out at a livestock farm of West Bengal University of Animal and Fishery Sciences, Belgachia, Kolkata, West Bengal. The farm data and samples from ducks were collected from March to May, 2020. Later, samples were processed for appropriate molecular biology work. The annual mean temperature is 26.8°C (80°F); monthly mean temperatures range from 19°C to 30°C (67°F to 86°F), and maximum temperatures can often exceed 40°C (104°F) during May–June.

In total, 60 healthy indigenous ducklings (*Anas platyrhynchos*) were used for this study. The ducklings were properly maintained under standard feeding and management. Duck eggs were collected from the field and subjected to hatching for an average of 28 days and reared in our farm from day-old stage. The ducklings (day old to 2 weeks of age) were given a starter ration containing 20% crude protein and 2,750 Kcal ME per kg. The first growing phases (3–8 weeks) received the feed composed of 18% crude protein and 2,750 kcal ME per kg, and the second growing phases (9–20 weeks) received the feed composed of 15% crude protein and 2,700 kcal ME per kg (22). Studies were conducted with ethical approvals obtained from the Institutional Animal Ethics Committee, West Bengal University of Animal and Fishery Sciences.

### Data and sample collection

At the end of each week, the ducks were weighed using a digital weighing balance to determine the body weight in grams. Other morphometric parameters, i.e., shank length, breast width, keel length, drumstick length, neck length, body length, chest girth, and wing length, were measured with measuring tape for 8 weeks and expressed in centimeters (cm). Daily body weight gain was assessed through a computational approach.


$$\begin{array}{l} \text{Daily body weight gain}=\text{Body weight}/\text{age of the bird}. \end{array}$$

Based on the available data, the entire duckling population data are classified into high body weight (better growing) as values above mean + standard deviation, and low body weight (less growing) as values less than mean – standard deviation. Trends for average daily body weight gain have been depicted. Similar groups were also observed for average daily body weight gain and biomorphometric traits of the ducks.

Duodenum and cecum were collected from the duck belonging to the two groups, namely, higher and lower body weights. As a routine farm operation, at the laying stage, we maintain male:female as 1:10, and we sell the surplus males. It was clearly mentioned that based on the available data, the entire duckling population data were classified into *high body weight (better growing*) as values above mean + standard deviation and *low body weight (less growing)* as values less than mean – standard deviation.

Six ducks (males) from each group, *high body weight* and *low body weight*, were sold as a routine farm operation. Individual birds were already tagged at a very early age of their life. Samples of duodenum and cecum were collected in TRIzol maintaining cold chain and other norms from the slaughterhouse from the same birds (already tagged) sold to them.

### Characterization of mucin 2 gene in *Anas platyrhynchos*

All experiments were conducted in accordance with relevant guidelines and regulations of the Institutional Animal Ethics Committee, and all experimental protocols were approved by the Institutional Biosafety Committee, West Bengal University of Animal and Fishery Sciences, Kolkata.

The total RNA was isolated from the duodenum and cecum of Duck by TRIzol method (15, 23–26) and was further used for cDNA synthesis.

### Materials

Taq DNA polymerase, 10× buffer, and dNTP were purchased from Invitrogen, and SYBR Green qPCR Master Mix (2× ) was obtained from Thermo Fisher Scientific Inc. (PA, USA). L-Glutamine (Glutamax 100× ) was purchased from Invitrogen Corp. (Carlsbad, CA, USA). Penicillin-G and streptomycin were obtained from Amresco (Solon, OH, USA). Filters (Millex GV. 0.22 μm) were purchased from Millipore Pvt. Ltd. (Billerica, MA, USA). All other reagents were of analytical grade.

### Synthesis, confirmation of cDNA, and PCR amplification of mucin 2 gene

The 20 μl reaction mixture contained 5 μg of total RNA, 0.5 μg of oligo dT primer (16–18 mer), 40 U of ribonuclease inhibitor, 10 M of dNTP mix, 10 mM of DTT, and 5 U of MuMLV reverse transcriptase in reverse transcriptase buffer. The reaction mixture was gently mixed and incubated at 37°C for 1 h. The reaction was stopped by heating the mixture at 70°C for 10 min and chilled on ice. The integrity of the cDNA was checked by PCR. To amplify the full-length open reading frame (ORF) of gene sequence, a specific primers pair was designed based on the mRNA sequences of *Gallus gallus* by DNASTAR software. The primers have been listed in Table 1; 25 μl of the reaction mixture contained 80–100 ng cDNA, 3.0 μl of 10× PCR assay buffer, 0.5 μl of 10 mM dNTP, 1 U Taq DNA polymerase, 60 ng of each primer (as in Table 1), and 2 mM MgCl_2_. PCRs were carried out in a thermocycler (PTC-200, MJ Research, USA) with cycling conditions such as initial denaturation at 94°C for 3 min, denaturation at 94°C for 30 s, varying annealing temperature (as mentioned in Table 1) for 35 s, and extension at 72°C for 3 min for 35 cycles followed by a final extension at 72°C for 10 min.

**Table 1A:** PCR primers for mucin 2 gene characterization in duck.

		**Product length**	**Annealing temperature °C**
Duck mucin 1	FP: TGGAGTTCACAGTTCACCCA RP: TCGTCAAATGCAGAGGAGGT	726	55.0
Duck mucin 2	FP: TCAGCAAACCCAATGATGAA RP: TCCATCTGCCTGAATCACAG	552	55.2
Duck mucin 3	FP: TGTCCAGGCACATGTTCAGT RP: TTCCAGCTTTTCTGCTTGGT	448	55.3
Duck mucin 4	FP: ACCAAGCAGAAAAGCTGGAA RP: ACACCGGATCTTTGCATTTC	585	54.6
Duck mucin 5	FP: GAAATGCAAAGATCCGGTGT RP: TTAATGGCCTTTGAGCAGGT	544	55.5
Duck mucin 6	FP: ACAGAGAACGTCCCTTGTGG RP: ATGCAGCAACAGCAGAACAG	583	55.9
Duck mucin 7	FP: CTGTTCTGCTGTTGCTGCAT RP: CAGCGTCACAAGGAATTTCA	950	55.4
Duck mucin 8	FP: AATTCCTTGTGACGCTGGAC RP: GAGGACGTGTTACGGAGGTG	950	55.5
Duck mucin 9	FP: ACTCCTGAGAGCACAACACG RP: GTGGTAGTGGGGGCAGAC	713	54.4
Duck mucin 10	FP: CATCGGCAGCACCGTGAGCA RP: CGGAGGTGGTGGGTGTTGGC	851	55.3
Duck mucin 11	FP: CCTCCAGCAGCACCATCGGC RP: ATCCAGTTGGCGTTGGCGGG	843	55.5
Duck mucin 12	FP: ACAACGCCCTCCAGCAGCAC RP: GCCACCATGACTCACCCGGC	916	55.5
Duck mucin 13	FP: ATGACTGCAGGTTGCCAAATGGA RP: ACAGGACAGCACTCATCACTGGGA	838	53.5
Duck mucin 14	FP: ACAAGTGTGTTCCCAAGAAGGTTTGT RP: ACAGGATACTGTAGCGGGGAAAAGC	925	52.5

**Table 1B:** PCR primers quantitative PCR for mucin 2 gene in duck.

	**PCR primers used for QPCR**	**Annealing temperature, °C**
Mucin gene	FP: 5'-TCACCCTCATATACTTCTCA-3' RP: 5'-TTCCATCTCCTAATCACAT-3'	60
GAPDH gene (housekeeping)	FP: 5'-ATGTTCGTGATGGGTGTGAA-3' RP: 5'-CTGTCTTCGTGTGTGGCTGT-3'	

### cDNA cloning and sequencing

PCR amplicons verified by 1% agarose gel electrophoresis (Supplementary Figures) were purified from gel using a gel extraction kit (Qiagen GmbH, Hilden, Germany) and ligated into a pGEM-T easy cloning vector (Promega, Madison, WI, USA) following the manufacturer's instructions. The 10 μl of the ligated product was directly added to 200 μl competent cells, and heat shock was given at 42°C for 45 s in a water bath, and cells were then immediately transferred on chilled ice for 5 min, and SOC was added. The bacterial culture was pelleted and plated on an LB agar plate containing ampicillin (100 mg/ml) added to the agar plate 1:1,000, IPTG (200 mg/ml), and X-Gal (20 mg/ml) for blue-white screening. Plasmid isolation from overnight-grown culture was done by the small-scale alkaline lysis method. Recombinant plasmids were characterized by PCR using gene-specific primers and restriction enzyme digestion based on the reported nucleotide sequence for chicken. The enzyme EcoRI (MBI Fermentas, USA) is used for fragment release. Gene fragment inserts in the recombinant plasmid were sequenced by an automated sequencer (ABI prism) using the dideoxy chain termination method with T7 and SP6 primers (12, 18, 20, 27–32).

### Sequence analysis

The nucleotide sequence so obtained was analyzed for protein translation, sequence alignments, and contigs comparisons by DNASTAR Version 4.0, Inc., USA. The novel sequence was submitted to the NCBI Genbank, and an accession number was obtained which is available in the public domain now.

### Study of predicted mucin 2 peptide using bioinformatics tools

The predicted peptide sequence of Mucin 2 of indigenous duck was derived by Edit sequence (Lasergene Software, DNASTAR) and then aligned with the peptide of other chicken breeds and avian species using Megalign Sequence Programme of Lasergene Software, DNASTAR (12, 14, 18, 20, 27–29, 31–35).

Prediction of the signal peptide of the Mucin 2 gene was conducted using the software (Signal P 3.0 Sewer-prediction results, Technical University of Denmark). Estimation of leucine percentage was conducted manually from the predicted peptide sequence. Disulfide bonds were predicted using suitable software (http://bioinformatics.bc.edu/clotelab/DiANNA/) and by homology search with other species.

A protein sequence-level analysis study was carried out with specific software (http://www.expasy.org./tools/blast/) for the determination of leucine-rich repeats (LRR), leucine zipper, N-linked glycosylation sites, detection of leucine-rich nuclear export signals (NES), and detection of the position of GPI anchor. Detection of leucine-rich nuclear export signals (NES) was carried out with the NetNES 1.1 server, Technical University of Denmark (35). Analysis of O-linked glycosylation sites was carried out using the NetOGlyc 4 server (http://www.expassy.org/), whereas the N-linked glycosylation site was detected by the NetNGlyc 1.0 software (http://www.expassy.org/) (36). Detection of leucine-zipper was conducted through the Expassy software, Technical University of Denmark (37). Regions for alpha-helix and beta-sheet were predicted using NetSurfP (38). Protein Surface Accessibility and Secondary Structure Predictions, were analyzed as per the Technical University of Denmark. Domain linker prediction was done according to the software developed (39). LPS-binding sites (40) and LPS-signaling sites (41) were predicted based on homology studies with polypeptides of other species.

### Three-dimensional structure prediction and model quality assessment

The templates that possessed the highest sequence identity with our target template were identified by using PSI-BLAST (http://blast.ncbi.nlm.nih.gov/Blast). The homology modeling was used to build a 3D structure based on homologous template structures using the PHYRE2 server (42). The 3D structures were visualized by PyMOL (http://www.pymol.org/), which is an open-source molecular visualization tool. Subsequently, the mutant model was generated using the PyMoL tool. The Swiss PDB Viewer was employed for controlling energy minimization. The structural evaluation along with a stereochemical quality assessment of the predicted model was carried out by using SAVES (Structural Analysis and Verification Server), which is an integrated server (http://nihserver.mbi.ucla.edu/SAVES/). The ProSA (Protein Structure Analysis) webserver (https://prosa.services.came.sbg.ac.at/prosa) was used for the refinement and validation of protein structure (43). The ProSA was used for checking model structural quality with potential errors, and the program shows a plot of its residue energies and Z-scores, which determine the overall quality of the model. The solvent accessibility surface area of the mucin 2 gene was generated by using the NetSurfP server [http://www.cbs.dtu.dk/services/NetSurfP/; (38)]. It calculates the relative surface accessibility, Z-fit score, the probability for alpha-helix, the probability for beta-strand and coil score, etc. TM align software was used for the alignment of 3D structure of IR protein for different species and RMSD estimation to assess the structural differentiation (44). PDB structure for 3D structural prediction of the mucin 2 gene for the duck was carried out through the PHYRE software. Protein–protein interactions have been studied through the String analysis (45).

### Real-time PCR

Total RNA was estimated from the duodenum and cecum of duck from high-body weight and low-body weight groups by the TRIzol method, and the quantitative analysis of total RNA was performed using formaldehyde gel electrophoresis. The 28S rRNA and 18S rRNA demarcated the quality of RNA. First-strand cDNA was synthesized by the process of reverse transcriptase polymerase chain reaction (RT-PCR) in the automated temperature-maintained thermocycler machine. M-MLVRT (200 u/μl) was used as a reverse transcriptase enzyme. All the primers were designed using the primer 3 software (v. 0. 4.0) as per the recommended criteria. The primers used are listed in Table 1. An equal amount of RNA (quantified by Qubit fluorometer, Invitrogen), wherever applicable, was used for cDNA preparation (Superscript III cDNA Synthesis Kit; Invitrogen). All qRT-PCRs were conducted on ABI 7500 fast system. Each reaction consisted of 2 μl cDNA template, 5 μl of 2× SYBR Green PCR Master Mix, 0.25 μl each of forward and reverse primers (10 pmol/μl), and nuclease-free water for a final volume of 10 μl. Each sample was run in duplicate. Analysis of real-time PCR (qRT-PCR) was performed by the delta–delta–Ct (ΔΔCt) method (32–34).

The entire reactions were performed in triplicate (as per MIQE Guidelines), and the experiment was repeated two times, in 20 μl reaction volume, using FastStart Essential DNA Green Master (Himedia) on ABI 7500 system.

### Histological section

The duodenum and cecum samples were fixed in formalin (10%), embedded in paraffin, and processed for histological examination and stereology. The tissues were submerged in Lillie fixative for 1 week at room temperature and then were processed and embedded vertically in paraffin wax. Then, each sample was exhaustively sectioned into 4 μm-thick sections by a fully automated rotary microtome (Leica RM2255, Germany). Each of these sections was stained with hematoxylin and eosin and mounted. From each sample, 10–15 sections were chosen by the systematic uniform random sampling (SURS) method.

### Immunohistochemistry

Paraffin tissue blocks were prepared by standard manual alcohol-acetone protocol. The 5–6 μm thick paraffin sections obtained from the duodenum of both the high-growth and low-growth ducks were taken on the Millennia 2.0 adhesion slides (Cat. No. 71863-01, Abcam). The tissue sections were de-paraffinized and hydrated in distilled water. The tissue slides were covered with trypsin enzymatic antigen-retrieval solution (Cat. No. ab970, Abcam) and kept in an incubator in a humid environment at 37°C for 5–10 min. The sections were then incubated for 60 min in peroxidase blocking solution (Lot. No. 00065614, Dako) at room temperature to block non-specific antibody-binding activity. After subsequent washing with phosphate-buffered saline (PBS), the sections were incubated at 37°C for 2 h in a humid environment with mouse monoclonal anti-mucin antibody in 1: 200 dilution. Immunoreactivity was detected after 1-h incubation at 37°C with a secondary antibody, Rabbit anti-mouse IgG H&L (HRP Conjugated, Cat. No. ab6728; Abcam) in dilution 1:200. Slides were then rinsed three times in PBS for 5 min each, followed by treatment with freshly prepared DAB solution for 3 min (DAB substrate, Cat. No. 34001, Thermo Fisher Scientific). The sections were counter-stained with Mayer's hematoxylin, hydrated in ethanol, cleared in xylene, and then mounted in DPX.

### Statistical analysis

Descriptive statistics with mean and standard error were estimated through the SYSTAT package for the expression level analyzed through real-time PCR and presented accordingly in the graph.

The model employed:


Yeijk=μ+Gi+Aj+eijk

Where, Y eijk = kth observation of the target trait, μ= overall mean, Gi = fixed effect of the ith genotype corresponding to the expression level, Aj = fixed effect of the jth bird, eijk = random error. Expression level with real-time PCR was estimated as 2^−Δ*ΔCt*^.

## Results

### Characterization of mucin gene in duck

Mucin 2 coats the epithelia of the intestines, airways, and other mucus membrane-containing organs. Certain important domains for mucin 2 protein have been detected. The total sequence length of the mucin 2 gene in *Anas platyrhynchos* is 12.013 KB. In this study, we could amplify the partial mucin 2 gene of Bengal duck as 11,968 bp, with gene bank accession number as OQ789085. In this study, we have reported mucin 2 gene sequence for ducks for the first time.

**Figure 1:**
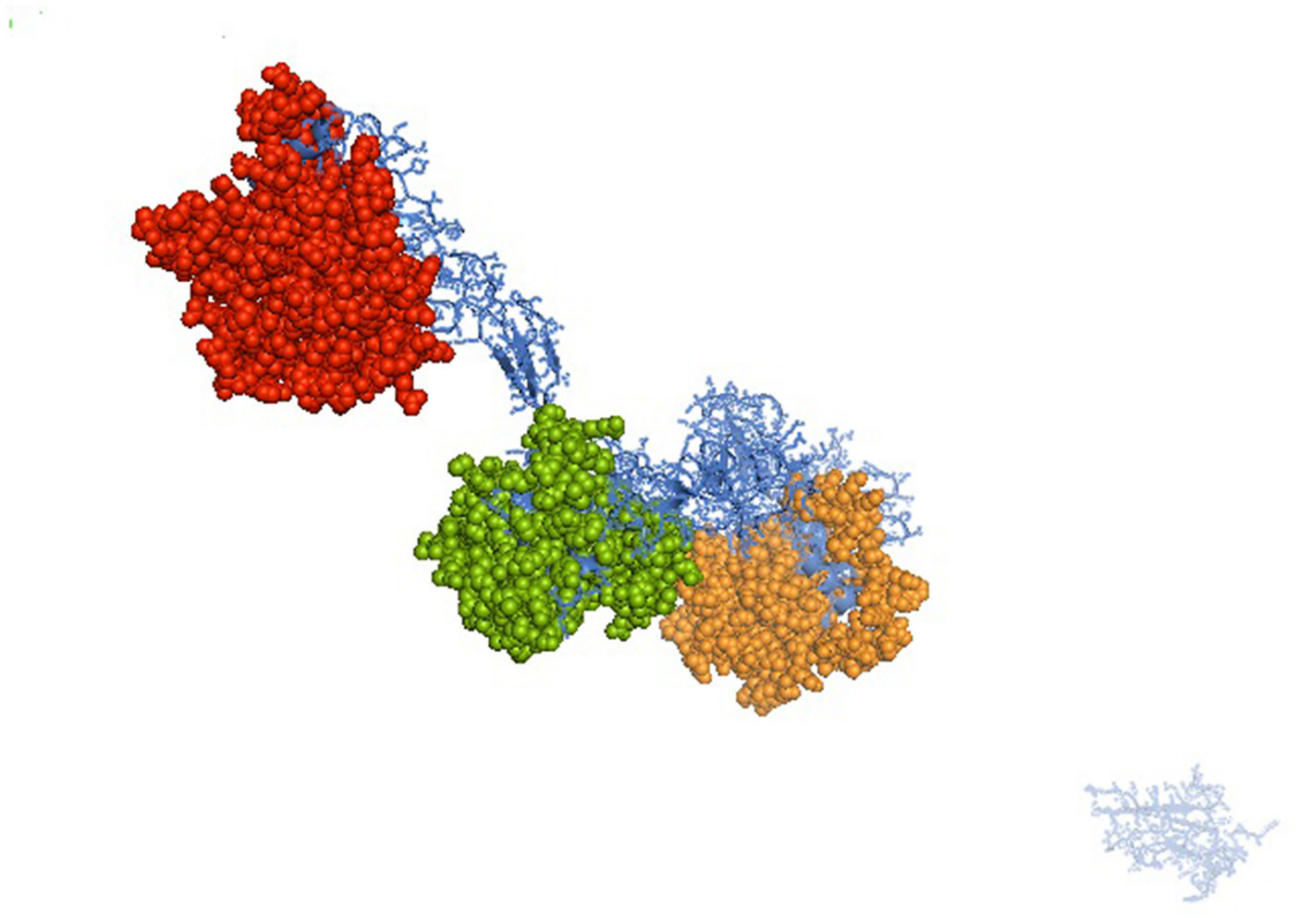
Predicted 3D structure for mucin 2 protein for Bengal duck. Identified domain for Mucin 2 – VWFD (von Willebrand factor) at amino acid sites of 33–236 (red sphere), 388–601 (forest green), 859–1,065 (orange sphere) for the mucin 2 gene of duck.

**Figure 2:**
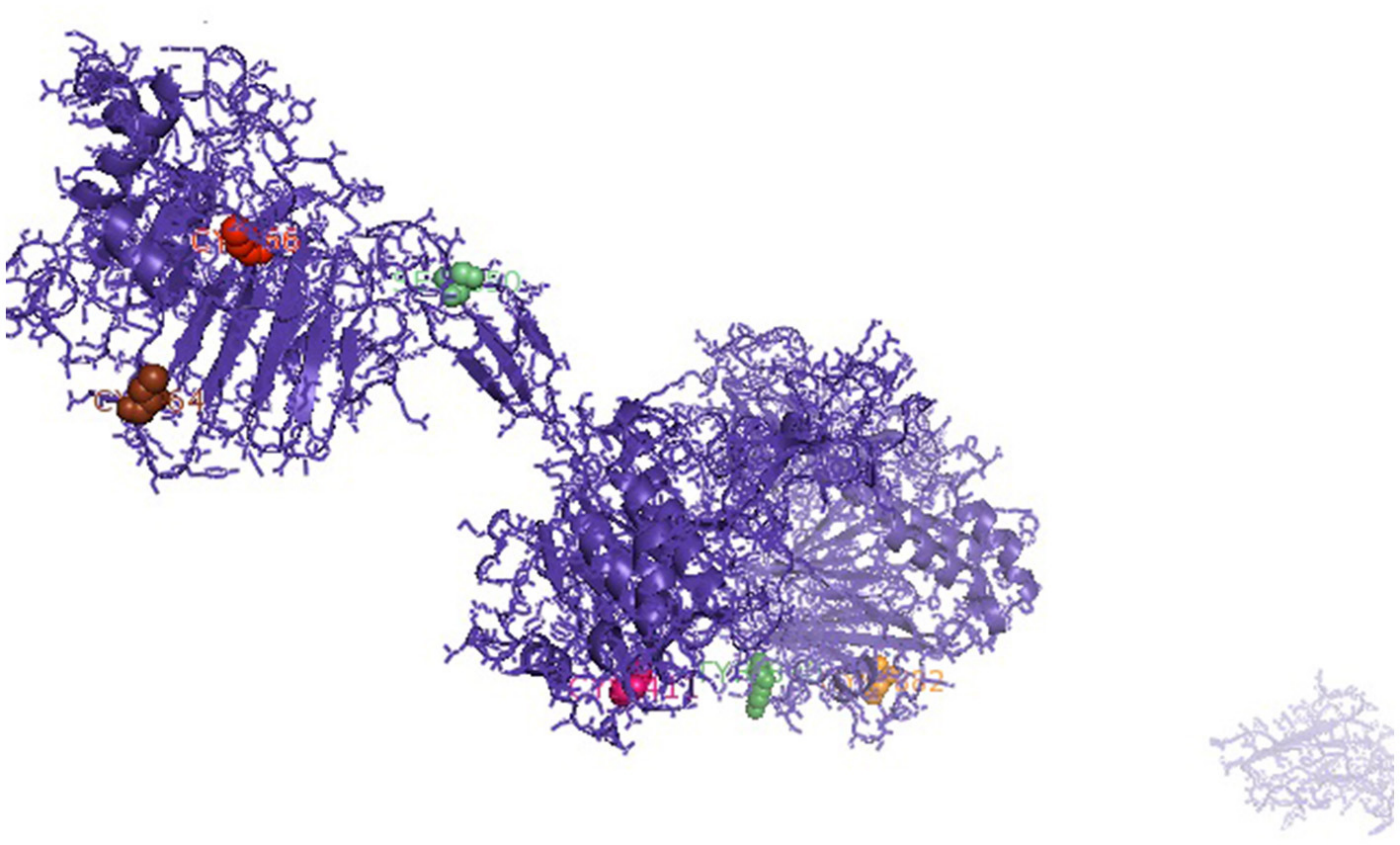
Sites for disulfide bonds were detected at aa positions 56–64, 411–419, 882–890, 2,968–2,976, 3,532–3,581, 3,557–3,611, and 3,561–3,613 (indicated in colored spheres) for the mucin 2 gene of duck.

It was attempted to explore the growth potential ability of the domains. The signal peptide was detected at amino acid position 1–18 for the predicted 3D protein structure of the mucin 2 gene. An important domain has been identified as the VWFD (von Willebrand factor domain) at amino acid sites of 33–236 (red sphere), 388–601 (forest green), 859–1,065 (orange sphere), and 2,945–3,164 (Figure 1). Other identified domains were VWFC-1 at sites of amino acids 3,293–3,343 and 3,402–3,448. VWFC-2 domains were predicted at the amino acid location of 3,275–3,344 and 3,382–3,449. Other two important sites were CTCK1 (C terminal cystine knot signature) and CTCK2 (C terminal cysteine knot domain profile). The sites for the domain of CTCK1 are 3,580–3,618 and CTCK2 at aa position 3,532–3,619. Sites for disulfide bonds were detected at aa positions 56–64, 411–419, 882–890, 2,968–2,976, 3,532–3,581, 3,557–3,611, and 3,561–3,613 (Figure 2).

In the current study, we are concerned with growth parameters for the mucin gene. We had identified an important domain involved with growth as EGF1 (EGF-like domain signature) at sites 3,296–3,307. Certain important observations were revealed. Since disulfide bonds were not included in this region, it is presumed to be open. EGF1 domain is inserted within VWFC-1 and VWFC-2.

The sites for glycosylation are very important. The sites for N-linked glycosylation and O-linked glycosylation have been listed in Supplementary material; 22 sites for N-linked glycosylation and O-linked glycosylation have been predicted.

**Figure 3:**
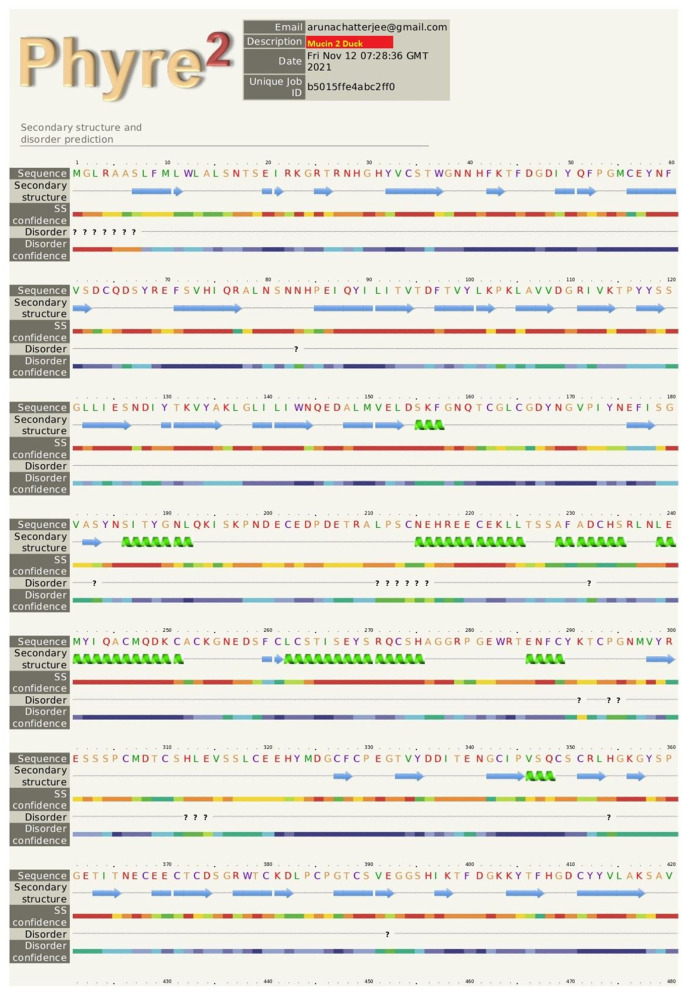
The pictorial description for secondary structure with the sites for alpha helix, beta sheet, and loop structure for the mucin 2 gene of duck. 
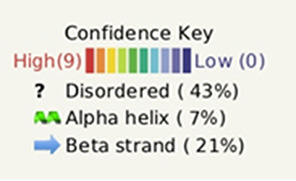

The pictorial description for the secondary structure of the mucin 2 gene is described in Figure 3. It represents the site for the alpha helix, beta sheet, and loop structure for the mucin gene.

### Growth parameters for Bengal duck

**Figure 4:**
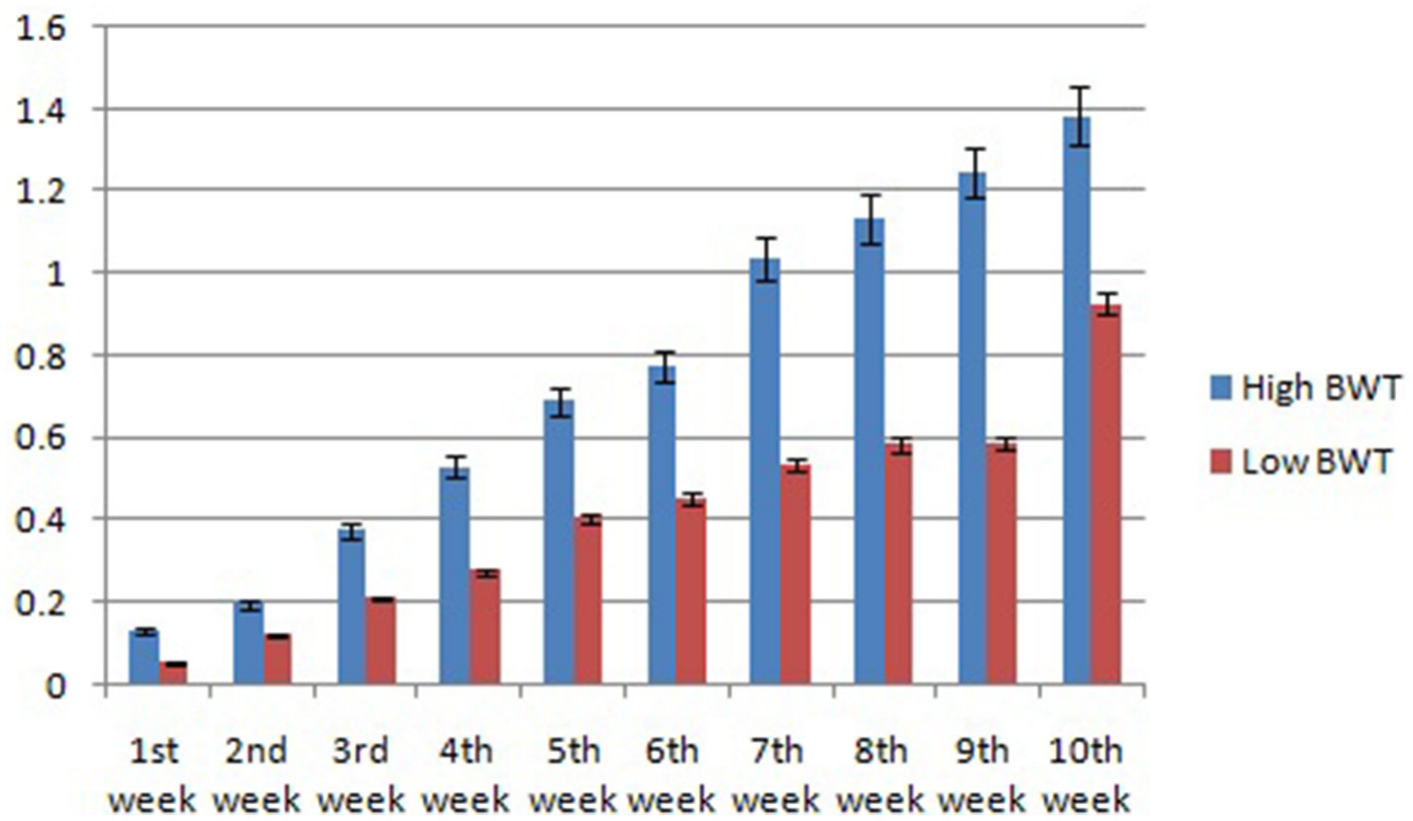
Graphical representation for body weight of indigenous ducks at successive stages of life.

**Figure 5:**
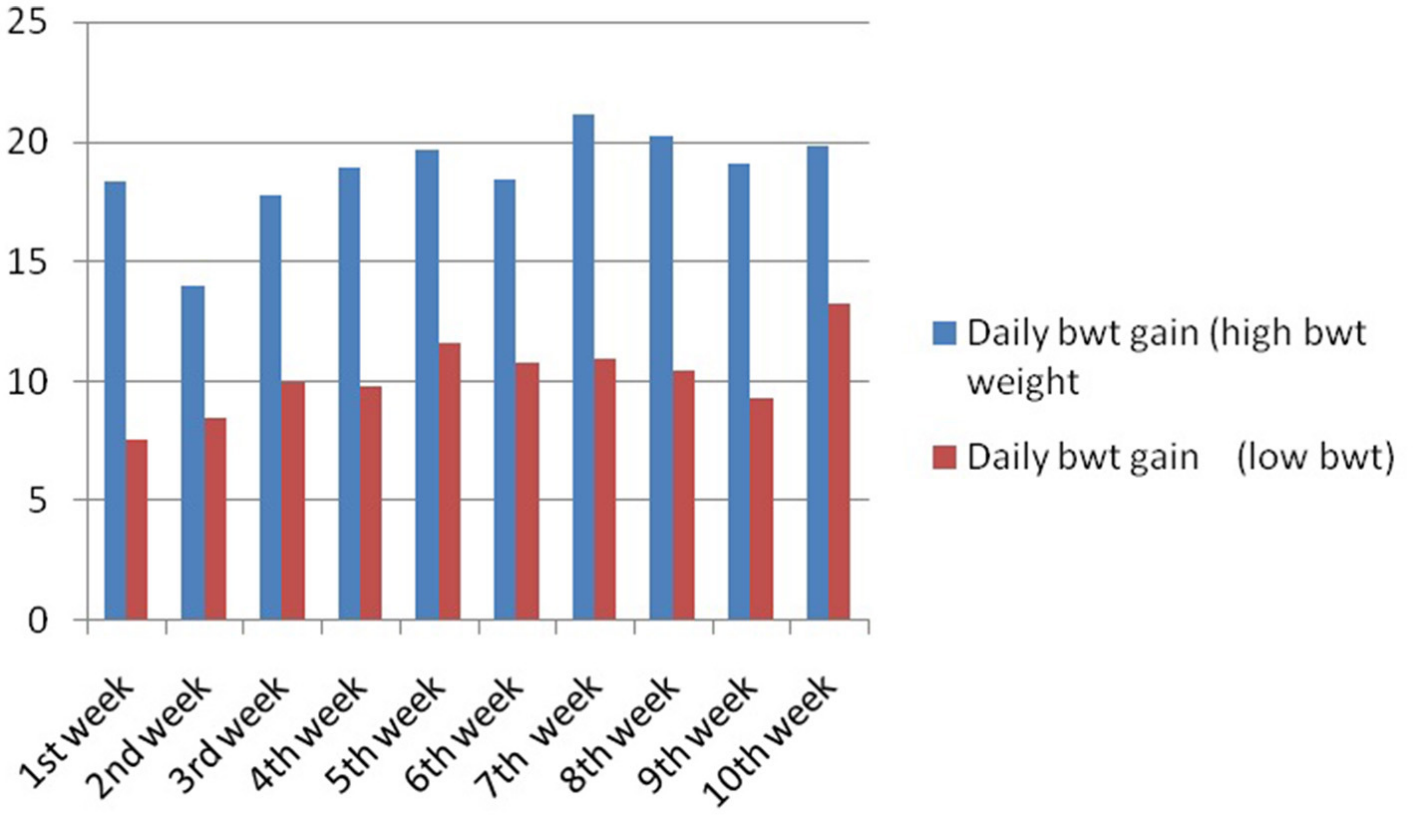
Daily body weight gain for Bengal duckling at successive ages.

In this study, we studied the growth parameters of the indigenous duck of Bengal – Bengal duck. Body weight was studied of the ducklings just after hatching up to the 10th week of age (Table 2). Figure 4 represents the diagrammatic representation of body weight at successive ages of brooding of Bengal ducks up to the 10th week of age. Accordingly, the average daily body weight gain was also recorded at successive ages up to the 10th week (Table 3). Figure 5 shows the diagrammatic representation of the average daily body weight gain for both the groups of ducklings – Group I (higher growth) and Group II (lower growth).

**Table 2:** Body weight at successive weeks of life for two different levels of growth.

**Age**	**Group I (High body weight, g)**	**Group II (Low body weight, g)**
1st week	128.6± 3.331^a^	53.0 ± 1.176^b^
2nd week	195.478 ± 5.751 ^a^	118.667 ± 1.153 ^b^
3rd week	373.3± 5.86 ^a^	209.0± 1.0522 ^b^
4th week	529.375 ± 7.074 ^a^	275.0± 2.0558 ^b^
5th week	688.9± 10.907 ^a^	406.571± 1.9236 ^b^
6th week	773.862± 11.091 ^a^	454.0± 3.4616 ^b^
7th week	1,035.6± 12.391 ^a^	534.778± 3.4354 ^b^
8th week	1,134.667± 13.924 ^a^	585.778± 4.0664 ^b^
9th week	1,246.0 ± 13.488 ^a^	588.0± 4.4806 ^b^
10th week	1,385.548± 14.245 ^a^	927.222± 7.259 ^b^

For all values, the level of significance P ≤ 0.01. ^a, b^ represent significant differences.

**Table 3:** Average daily body weight gain.

**Age**	**Group I (High body weight, g)**	**Group II (Low body weight, g)**	**Probability**
1st week	18.371± 0.93^a^	7.571± 0.32^b^	*P ≤* 0.01
2nd week	13.963 ±0.84^a^	8.476 ±0.36^b^	*P ≤* 0.10
3rd week	17.776 ±0.92^a^	9.952 ±0.39^b^	*P ≤* 0.05
4th week	18.906^**a**^±0.95^a^	9.821 ±0.38^b^	*P ≤* 0.01
5th week	19.683 ±0.99^a^	11.616 ±0.43^b^	*P ≤* 0.05
6th week	18.425 ±0.94^a^	10.809 ±0.41^b^	*P ≤* 0.05
7th week	21.135 ± 1.04^a^	10.914 ±0.43^b^	*P ≤* 0.01
8th week	20.262 ± 1.06^a^	10.460 ± 0.41^b^	*P ≤* 0.01
9th week	19.078 ± 0.96^a^	9.333 ± 0.37^b^	*P ≤* 0.01
10th week	19.794 ± 0.98^a^	13.246 ±0.49^b^	*P ≤* 0.10

### Biomorphometric estimation for duck grouped into higher and lower growth parameters

**Figure 6:**
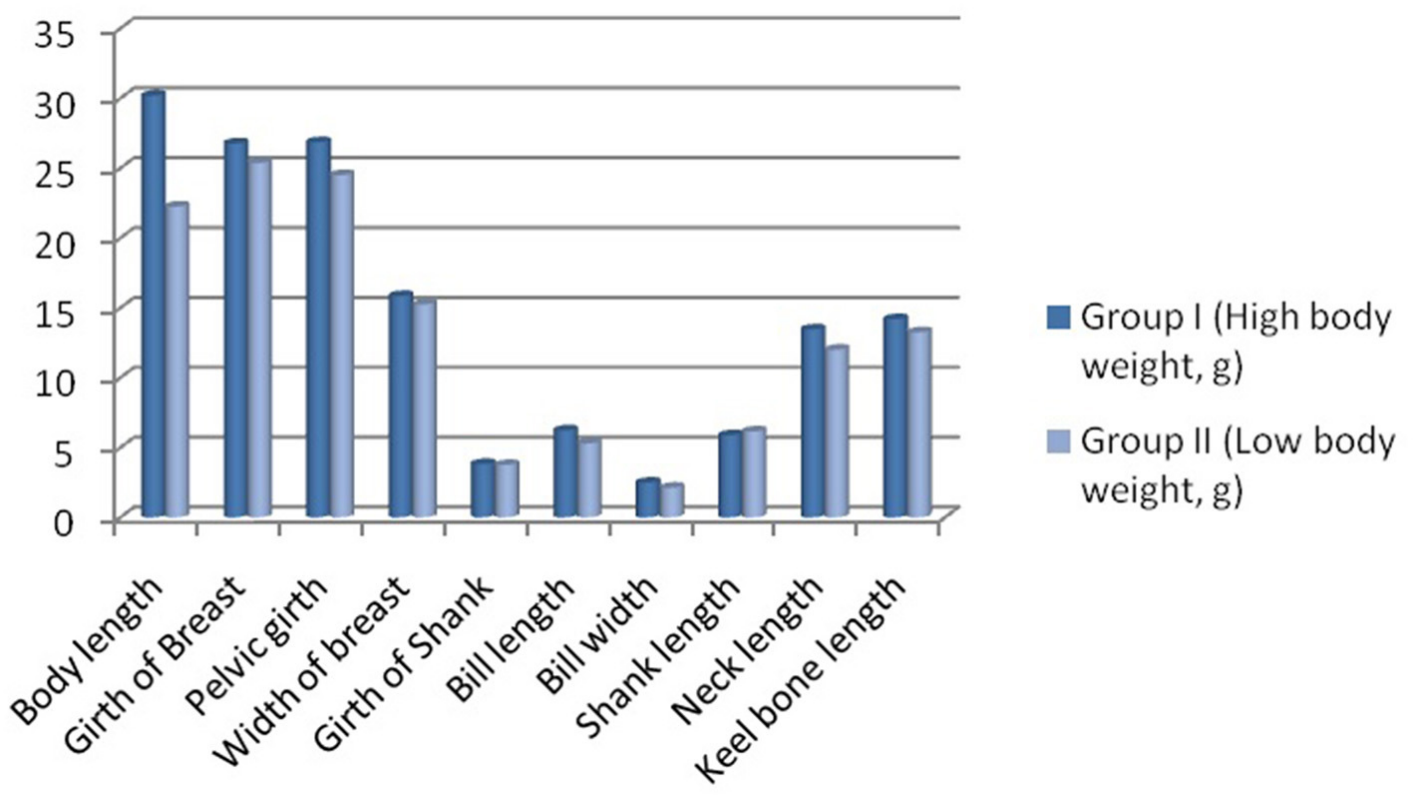
Biomorphometric characteristics for adult Bengal duck from Group I (higher growth) and Group II (lower growth).

Biomorphometric characteristics are useful parameters for assessing growth parameters. They also serve as an indirect way to assess the body weight of the birds. The average biometry characteristics for adult male and female ducks including pool average for both higher (Group I) and lower groups (Group II) have been listed in Table 4. Significant differences in growth parameters were observed for the body length of the Bengal duck. Figure 6 represents the morphometric characteristics of adult Bengal ducks from Group I (higher growth) and Group II (lower growth).

**Table 4:** Biomorphometric characteristics for Bengal duck.

	**Group I (High body weight, g)**	**Group II (Low body weight, g)**
Body length	30.19^a^ ± 1.043 ^a^	22.25^b^ ± 1.032 ^b^
Girth of Breast	26.76 ± 1.038	25.37± 1.035
Pelvic girth	26.85 ± 1.036	24.5 ± 1.033
Width of breast	15.85 ± 0.88	15.25 ± 0.86
Girth of Shank	3.82 ± 0.31	3.75 ± 0.28
Bill length	6.23± 0.64	5.3± 0.58
Bill width	2.48 ± 0.20	2.12 ± 0.18
Shank length	5.88 ± 0.56	6.15 ± 0.61
Neck length	13.46 ± 0.76	12 ± 0.75
Keel bone length	14.19 ± 0.83	13.25 ± 0.78

Superscripts a and b indicate significant differences at P ≤ 0.10.

**Figure 7:**
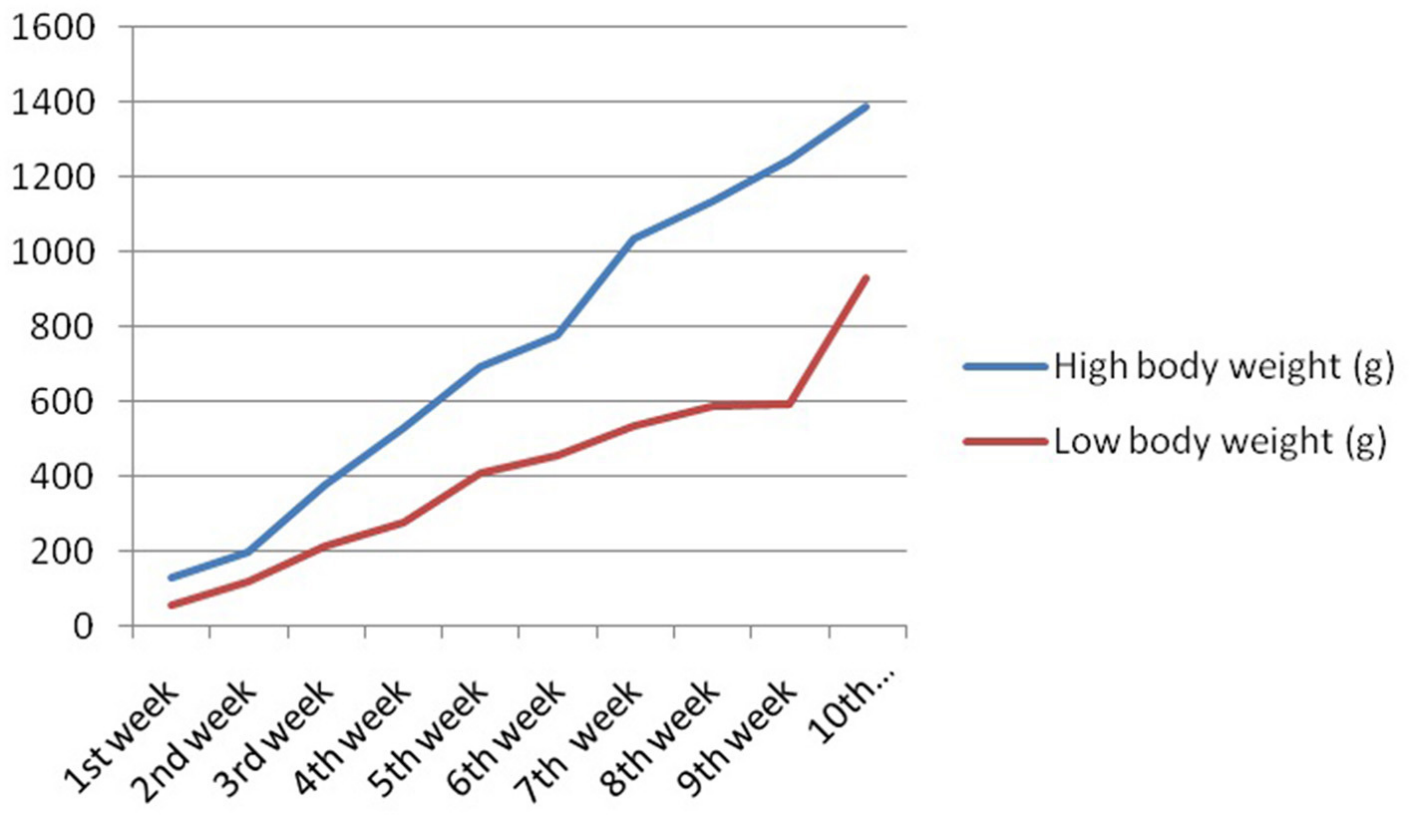
Growth curve for duck at successive stages of life based on body weight.

**Figure 8:**
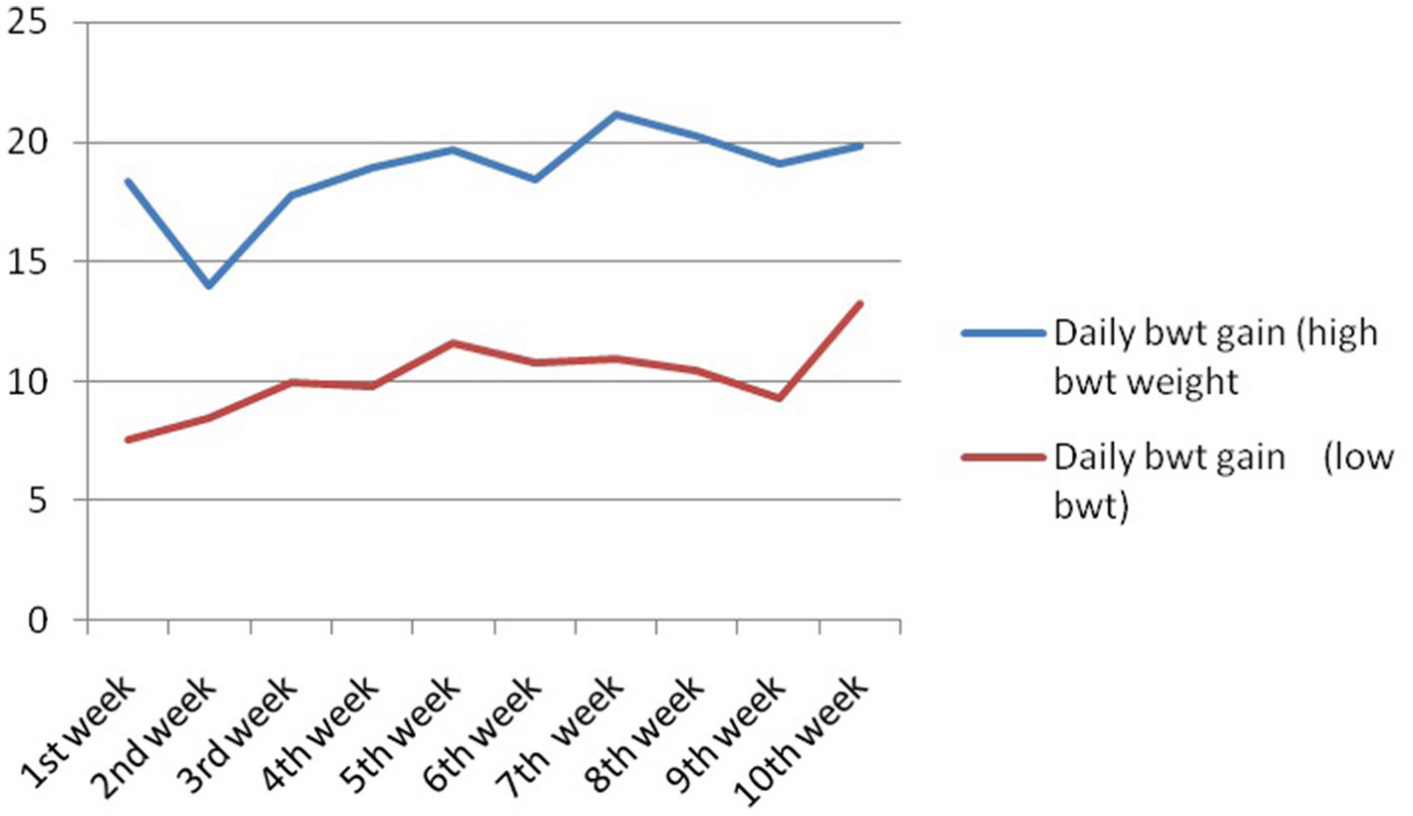
Growth trend representing daily body weight gain for Bengal duckling.

The growth curve for ducks at successive stages of life based on body weight is represented in Figure 7, whereas Figure 8 depicts the growth trend with respect to daily body weight gain.

### Expression profiling for mucin 2 genes with respect to growth parameters

Mucin is a protein expressed in the inner layer of the epithelium of some organs. It is commonly observed in the gut of livestock and poultry. In this study, we studied the expression profiling of two important organs – duodenum (part of small intestine) and cecum (part of large intestine) of Bengal ducks with respect to high- and low-body weights.

**Figure 9:**
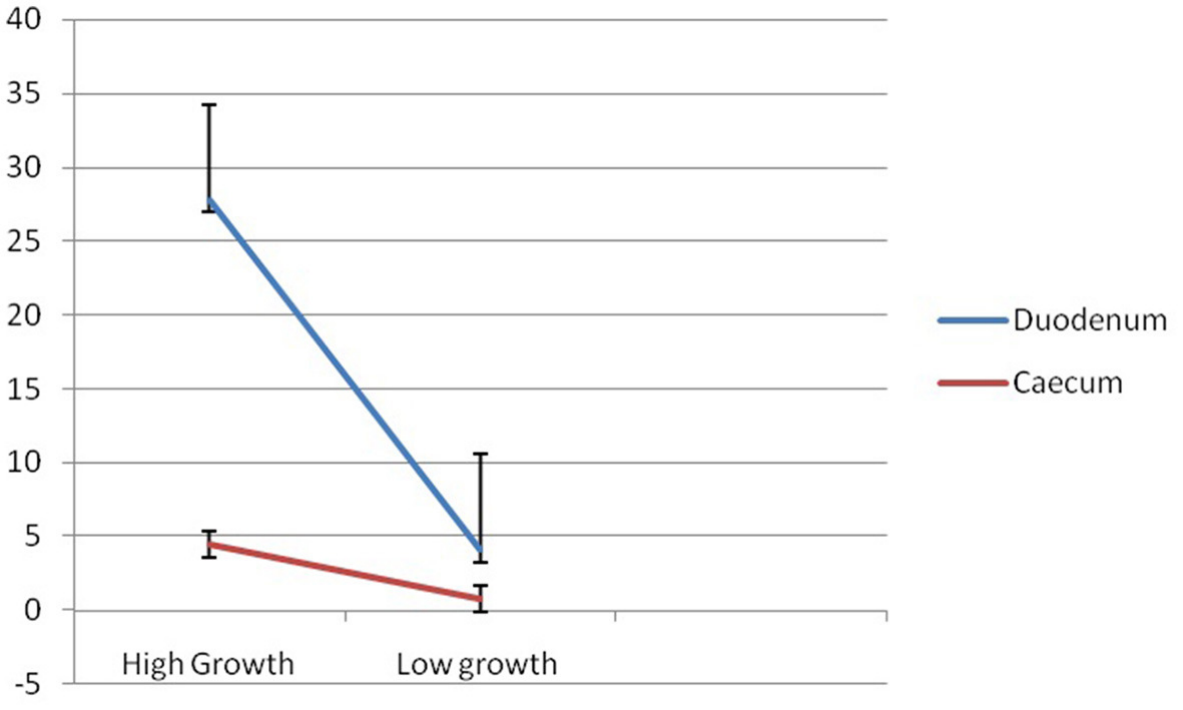
Differential mRNA expression profiling for Mucin 2 gene in duodenum and caecum of Bengal duck.

Figure 9 reflects the better expression profile for the mucin gene in Group I compared to that of Group II for duodenum and cecum, respectively. The expression for the mucin gene was observed to be more than 5-fold in Group I compared to that of Group II. It is evident from Figure 9 that although a similar trend of a better expression of mucin is observed for both the organs for the gut, mucin is better expressed in the duodenum compared to that of the cecum. The line graph represents a steep line in mucin expression in the duodenum, compared to that of the cecum. The expression of the mucin gene in Group I was observed to be more than 6-fold (6.29) in the duodenum compared to that of the caecum. Similarly, expression of mucin gene was observed to be more than 5-fold (5.3) in duodenum compared to that of cecum.

### Histological section and immunohistochemistry

**Figure 10:**
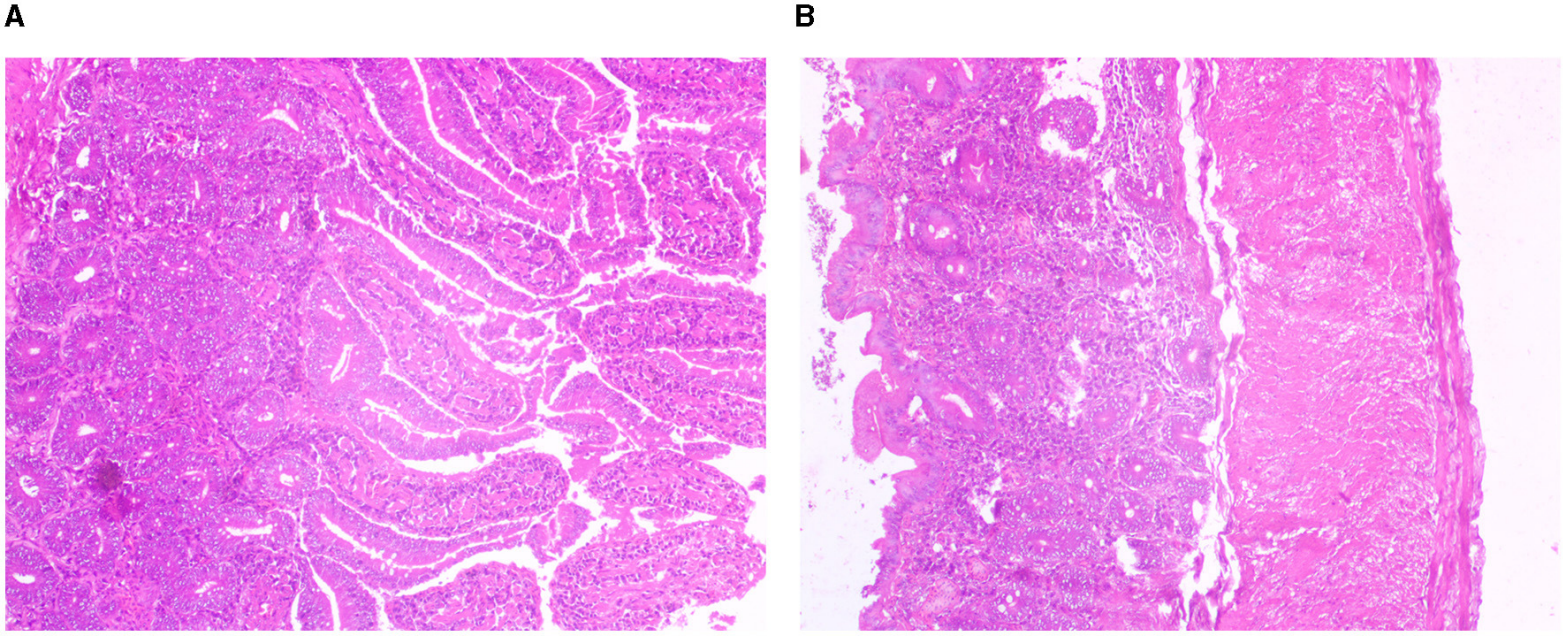
**(A)** Histological section for duodenum of Bengal duck. **(B)** Histological section for caecum for Bengal duck.

Light microscopic images of Mayer's hematoxylin-stained optical sections of the duodenum and cecum have been depicted in Figures 10A, B, respectively.

**Figure 11:**
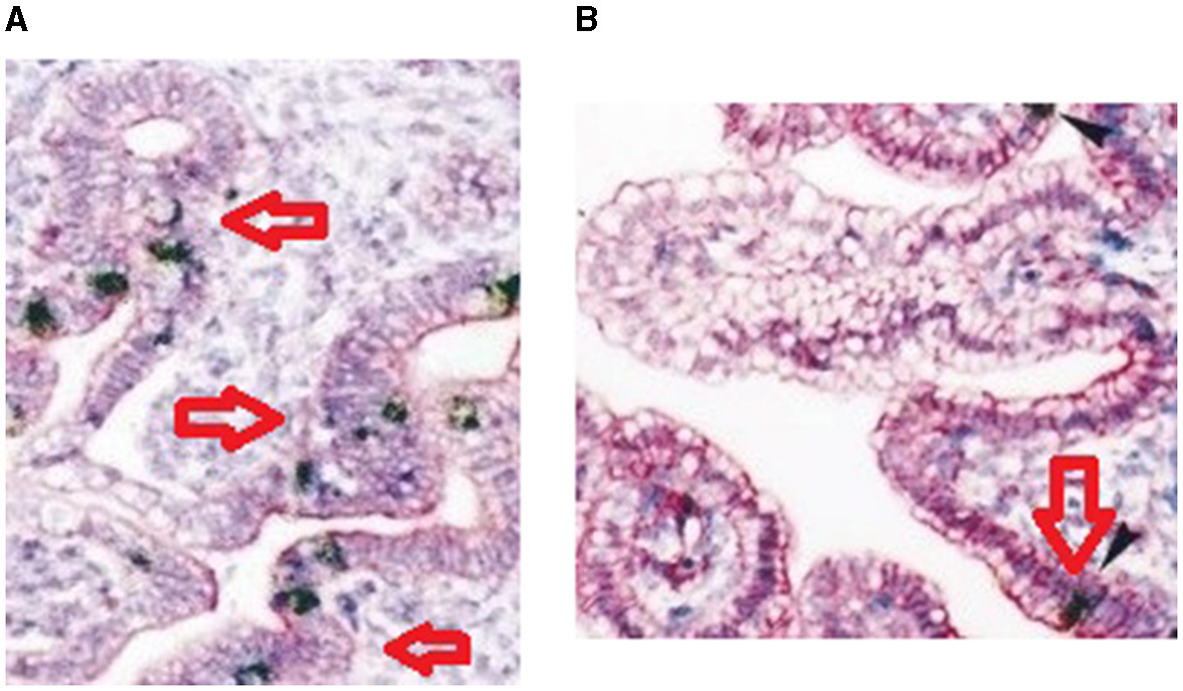
**(A)** Immunohistochemistry for duodenum of Bengal duck (high growth group). **(B)** Immunohistochemistry for duodenum of Bengal duck (low growth group).

Immunohistochemistry for the expression of mucin 2 protein in the duodenum of both the high-growth group and the low-growth group has been represented in Figures 11A, B, respectively.

## Discussion

The mucins are the main component of the mucus layer, produced and secreted by goblet cells. Mucin 2 gene from duck is sequenced and characterized here for the first time; hence, comparison was not possible. We observed certain important domains of duck mucin as VWFD, VWFC-1 and VWFC-2 domains, CTCK1 (C terminal cystine knot signature), and CTCK2 (C terminal cysteine knot domain profile). In this study, we aim to explore important domains for growth in mucin 2 gene of duck. Members of the C-terminal cysteine knot family are listed as von Willebrand factor (VWF), a multifunctional protein that is involved in maintaining homeostasis. It consists of four VWF type D domains, three VWF domains, two VWF type C domains. Since this is the first report of mucin 2 in ducks, we attempt to explore mucin 2 from other species with similar domains. Human mucin 2 is a highly polymorphic multidomain molecule with a modular architecture similar to VWF. Xenopus mucin B.1 contains a domain, an X domain, and a CTCK. Other mucins that contain a CTCK are the human tracheobronchial mucin (gene MUC5), bovine submaxillary apo mucin, and rat intestinal mucin-like protein. von Willebrand factor (VWF) is a large, multimeric blood glycoprotein synthesized in endothelial cells and megakaryocytes, which is required for normal hemostasis. The type D domain (VWFD) requires clotting factor VIII binding and normal multimerization of VWF (https://prosite.expasy.org/PDOC00928). The VWFC domain is essential for its function named after the von Willebrand factor (VWF) type C repeat which is found two times in this multidomain protein. It has a length of about 70 amino acids covering 10 well-conserved cysteines (https://prosite.expasy.org/rule/PRU00580). The duplicated VWFC domain is thought to participate in oligomerization, for Von Willebrand factor, but not necessary in the initial dimerization step. The presence of this region in other complex-forming proteins leads to the assumption that the VWFC domain might be involved in forming larger protein complexes.

We could identify an important domain involved with growth as EGF1 (EGF-like domain signature), presumed to play a major role in growth promotion. Certain important observations revealed that since disulfide bonds were not included in this region, it is presumed to be open. EGF1 domain is inserted within VWFC-1 and VWFC-2. These are presumed to affect growth traits (46–49). Other reports also reported similar observations that mucins are high-molecular-weight glycoproteins (50–80% O-linked oligosaccharides) produced by epithelial tissues in most animals. These glycoproteins are found in mucus (e.g., saliva and gastric juice) and secreted by mucous membranes to lubricate or protect the body surfaces of vertebrates, and the mucin is responsible for growth. In this study, we have confirmed our results. Further, they have a central role in maintaining epithelial homeostasis (1). Since this is the first report of studying mucin gene expression concerning growth parameters in any livestock species, comparison as such is not possible. However, there exists certain reports which depict that represented with histology followed by immunohistochemical studies. Similar confirmation of differential mRNA expression studies was also carried out by us earlier in case of CD8 and CD4 proteins in sheep model (50).

Other researchers have also explored the mode how mucin aid in growth. The mucus layer is part of the innate host response, protecting against luminal microflora, preventing gastrointestinal pathologies, and participating in the processes of nutrient digestion and absorption (2). A decrease in mucin synthesis in poultry could compromise the mucus layer and reduce nutrient utilization (3). In addition to its protective functions, mucin is involved in filtering nutrients in the gastrointestinal tract (GIT) and can influence nutrient digestion and absorption (11). Any component, dietary or environmental, that induces changes in mucin dynamics has the potential to affect viscosity, the integrity of the mucus layer, and nutrient absorption. In this study, we observed better expression of the mucin 2 gene in the duodenum in comparison to that of the cecum. Since it is evident that duodenum is greatly involved with the function of absorption of nutrients, better expression reveals that mucin 2 may be involved in nutrient absorption. Hence, it is evident that mucin has an active role in the process of nutrient digestion, absorption, and utilization. Thus, in the duck, the expression of less mucin indicates less nutrient content in the body, which will ultimately affect the growth. Mucins in general contain many threonine and serine residues, which are extensively O-glycosylated. Due to this profound glycosylation, mucins have a filamentous conformation. Reports are also available indicating mucin aids in threonine absorption (51). We observed high glycosylation of both O-linked and N-linked oligosaccharides. A certain set of reports indicates the role of threonine in growth. Debnath et al. (52) studied that threonine supplementation can positively influence antioxidant enzyme activities and hemato-biochemical parameters in commercial broilers, because threonine is one of the essential amino acids liable to be limited when high humidity and heat stress would decrease feed intake. Although no direct report is available about the mechanism of mucin in growth.

Certain reports are available for differential expression profiling of mucin genes in other aspects. It has been reported that probiotic supplementation increased the expression of the MUC2 gene in the chicken jejunum (53) and rat colon (54). Different factors such as microbial colonization in the intestine can affect the production, secretion, and composition of mucin (55, 56). Aliakbarpour et al. (57) studied that the inclusion of lactic acid bacteria-based probiotics in the diets significantly increased goblet cell number and villus length (*p* < 0.05) and significantly increased the gene expression (*p* < 0.05) with higher intestinal MUC2 mRNA in birds fed a diet with probiotics.

It has been observed that the body weight of the ducks at the brooding stage increases significantly from the lower body weight group of ducklings. However, the highest difference was observed at the first week of age. An increase of 58.78% was observed in the higher group in comparison to the lower body weight group. In the duckling of lower body weight group, from the 8th week to the 9th week, body weight was recorded to be similar. This is reflected as a decrease in daily body weight gain at the 9th week of age. It is interesting to note that a similar trend was observed for the ducklings of the higher body weight group. Average daily body weight was observed to be lowered at the 9th week of age. Since this is the first report on body weight estimation and rearing of ducklings of Bengal duck (indigenous duck of West Bengal), the comparison was not possible as such. We predict certain physiological changes in the duck at this stage of life, as the body diverts a part of nutrients for the development of reproductive organs.

Average daily body weight gain was observed to decrease at the second week of age in the higher body weight group. An increasing trend was observed up to the 5th week of age. To compare the growth performance of Bengal breeds of ducks with other available duck genetic resources, the average weekly live weight of Nageswari, Pekin, Muscovy, and Desi white ducks was collected from a published paper by Morduzzaman et al. (58) and Bhuiyan et al. (59). Body weight for Nageswari ducks was reported to be slightly more than Bengal ducks for ducklings from the successive week of age. This variation might be due to the differences in feed availability, nutrient content in the feed, management practices, and selection of the ducklings. Among these breeds including the desi duck of West Bengal, the highest body weight was produced by Pekin breeds of ducks (1,763 g) and the lowest in Desi white (1,208 g). Gonzalez and Marta (60) observed that the body weight of female khaki Campbell ducklings at 1, 4, and 7 weeks of age averaged 85.6, 585.1, and 1,113 g, while that of males were 75.0, 594.4, and 1,213.3 g for the same period. The body weight of White Pekin ducklings at the 6th week of age averaged 1,350 g and at the 7th week 1,718 g (61). In another investigation, Andrews et al. (62) reported that the adult body weight of desi ducks under the intensive and semi-intensive systems of rearing was 1,311 and 1,281 g, respectively.

The data on the mean daily body weight gain of indigenous ducks indicated that the body weight reached the highest in the 7th week, and after the 7th week, the body weight decreased slowly. The body weight at all ages was significantly (*p* < 0.05) different between generations. A higher mean value (11.4 g/bird) for daily body weight gain was recorded for those birds that fed wet mash than the birds that fed dry mash (63). Bale-Therik and Sabuna (64) reported that the bird fed with grit increased the feed intake, stimulated the digested enzyme, and improved the body weight. There was an average daily growth rate of 5.88 ± 0.05 and 6.27 ± 0.09 g per bird per day at their 8th week's growth phases, respectively (65).

But certain significant variations were detected in the phenotypic data for growth at different ages of duck. In the next step, we attempt to explore if any quantitative variations are present in the mucin gene. This quantitative expression profiling may arise due to the promoter region of the gene.

## Conclusion

It can be concluded that genetic variability has been observed among the growth traits of duck, and mucin gene has been observed to play a role in growth characteristics. Hence, mucin gene may be regarded as a promising gene affecting growth traits for indigenous duck, Bengal duck. This is the first report on the role of mucin gene in affecting growth traits.

Future studies must involve the validation of these works with more number of ducks. Mucin gene may be regarded as an important gene affecting the growth traits. Marker-assisted selection or genomic selection possess the future scope of the study. Other similar genes may be studied for growth traits for a better conclusive result.

## Data availability statement

The datasets presented in this study can be found in online repositories. The names of the repository/repositories and accession number(s) can be found in the article/Supplementary material.

## Ethics statement

The animal study was reviewed and approved by the Institutional Ethics Committee, West Bengal University of Animal and Fishery Sciences.

## Author contributions

AK, AP, and RS have designed the experiment and drafted the manuscript. AK, AP, MD, AC, SP, SB, AP, and UK have conducted the experiment. AbP have conducted bioinformatics analysis. AP, NG, and RS have reviewed the manuscript. All authors contributed to the article and approved the submitted version.
