# Supplementary material for: Role of mucin 2 gene for growth in Anas platyrhynchos: a novel report
Source: Front Vet Sci. 2023 Nov 9;10:1089451. doi: 10.3389/fvets.2023.1089451 (PMC10666069; doi:10.3389/fvets.2023.1089451)
Supplement: Supplementary file 1 [file Data_Sheet_1.pdf]

Supplementary Figures:The standard curve, with Ct value available in our system(ABI7500).

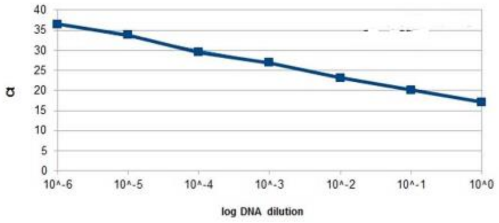

Amplification efficiency has been calculated from the slope

Y=-3.2746x+39.82

and correlation co-efficient R^2= 0.9974.

Please find other relevant graphs indicating the efficiency for QPCR :

Figure: Melting curve and melting peak for Mucin gene and house keeping gene (GAPDH)

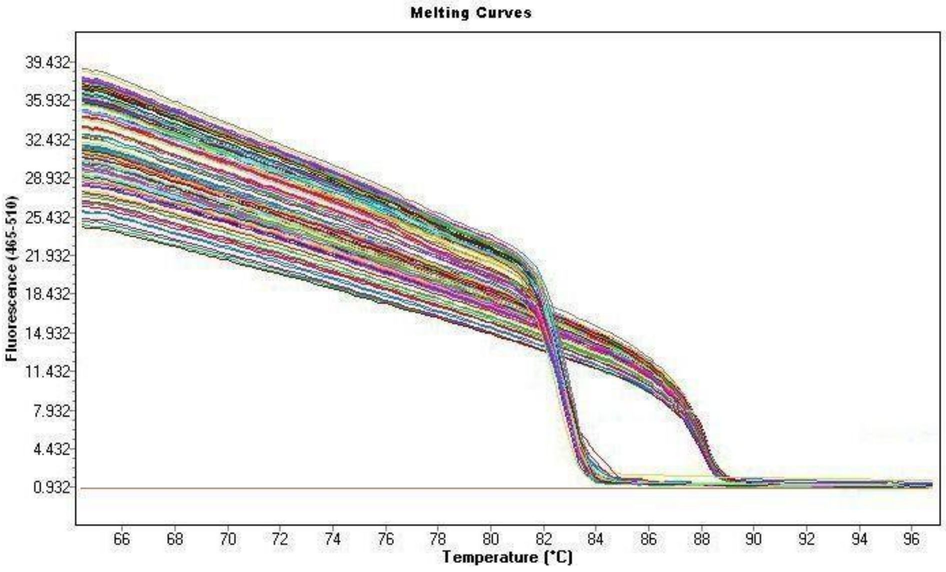

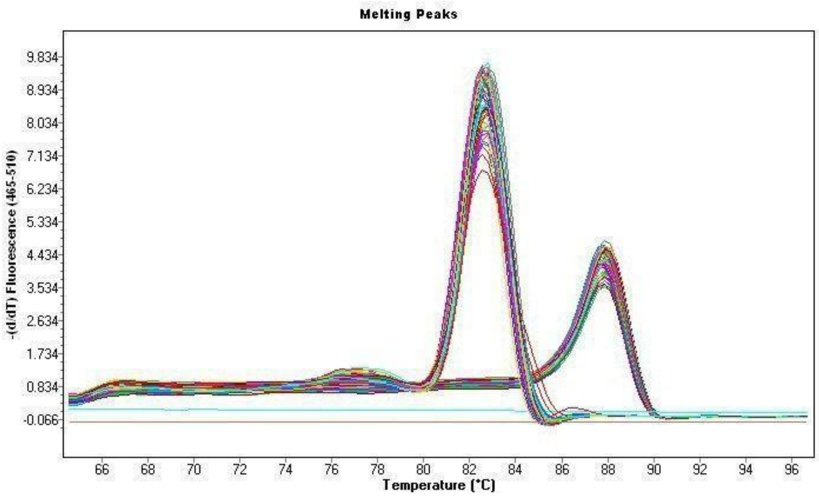

Melting Peaks
-(d/dT) Fluorescence (465-510)
9.834
8.934
8.034
7.134
6.234
5.334
4.434
3.534
2.634
1.734
0.834
-0.066
66
68
70
72
74
76
78
80
82
84
86
88
90
92
94
96
Temperature (°C)
